# Modular-integrative modeling: a new framework for building brain models that blend biological realism and functional performance

**DOI:** 10.1093/nsr/nwad318

**Published:** 2023-12-20

**Authors:** Mario Senden, Sacha J van Albada, Giovanni Pezzulo, Egidio Falotico, Ibrahim Hashim, Alexander Kroner, Anno C Kurth, Pablo Lanillos, Vaishnavi Narayanan, Cyriel Pennartz, Mihai A Petrovici, Lea Steffen, Tonio Weidler, Rainer Goebel

**Affiliations:** Department of Cognitive Neuroscience, Maastricht University, The Netherlands; Maastricht Brain Imaging Centre, Maastricht University, The Netherlands; Institute of Neuroscience and Medicine (INM-6) and Institute for Advanced Simulation (IAS-6) and JARA-Institut Brain Structure-Function Relationships (INM-10), Jülich Research Center, Germany; Institute of Zoology, University of Cologne, Germany; Institute of Cognitive Sciences and Technologies, National Research Council, Italy; The BioRobotics Institute, Scuola Superiore Sant’Anna, Italy; Department of Cognitive Neuroscience, Maastricht University, The Netherlands; Maastricht Brain Imaging Centre, Maastricht University, The Netherlands; Department of Cognitive Neuroscience, Maastricht University, The Netherlands; Maastricht Brain Imaging Centre, Maastricht University, The Netherlands; Institute of Neuroscience and Medicine (INM-6) and Institute for Advanced Simulation (IAS-6) and JARA-Institut Brain Structure-Function Relationships (INM-10), Jülich Research Center, Germany; RWTH Aachen University, Germany; Donders Institute for Brain, Cognition and Behavior, Radboud University, The Netherlands; Department of Cognitive Neuroscience, Maastricht University, The Netherlands; Maastricht Brain Imaging Centre, Maastricht University, The Netherlands; Cognitive and Systems Neuroscience Group, Swammerdam Institute for Life Sciences, University of Amsterdam, The Netherlands; Department of Physiology, University of Bern, Switzerland; FZI Research Center of Information Technology, Germany; Department of Cognitive Neuroscience, Maastricht University, The Netherlands; Maastricht Brain Imaging Centre, Maastricht University, The Netherlands; Department of Cognitive Neuroscience, Maastricht University, The Netherlands; Maastricht Brain Imaging Centre, Maastricht University, The Netherlands

## Abstract

This Perspective presents the Modular-Integrative Modeling approach, a novel framework in neuroscience for developing brain models that blend biological realism with functional performance to provide a holistic view on brain function in interaction with the body and environment.

To cope with the immense complexity of the brain, neuroscientific research often focuses on isolated brain structures or circumscribed perceptual, cognitive or motor functions [1]. This reductionist approach overlooks the intricate and multifaceted interplay among various structural and functional components. For instance, even simple movements require the coordinated activation of numerous neuronal populations across multiple brain regions [2]. Furthermore, and importantly, this approach ignores the complex interactions between the brain, the body and the environment, despite the significant role the latter two play in shaping cognition and perception [3].

Over the past decades, there has been a growing recognition that integrative brain models that synthesize various structural and functional subsystems will play an indispensable role in providing a more holistic understanding of the brain and its relation to the body and environment [4,5]. These models are large scale in the dual sense that they encompass various structural and functional components that, in turn, comprise large numbers of elementary units. The extent to which these elementary units need to reflect biological detail or overarching neurocomputational principles to achieve brain-like behavior in the integrated models is a point of contention among researchers [6]. This debate underscores the diverse methodologies in computational modeling, often characterized as ‘bottom-up’ and ‘top-down’ approaches. Bottom-up modeling emphasizes simulation of the detailed biological processes occurring in the brain [4,5]. Model parameters are primarily informed by existing biological data with the aim of providing meaningful constraints for functional capacities, which are assumed to spontaneously emerge [4,6]. Top-down modeling, by contrast, starts explicitly from functional capacities. It traditionally begins by identifying the functions of brain structures and then develops neurocomputational algorithms that realize these functions. In recent years, this hypothesis-driven approach has been supplemented with goal-driven deep learning [7]. This artificial intelligence–enabled approach aims to generate neurocomputational algorithms that realize brain function through parameter optimization such that the model can solve ecologically valid tasks [8]. Top-down models aim to achieve brain-like functionalities by emulating the brain’s overarching principles without necessarily simulating their biological details. For an in-depth review of these approaches, we refer the interested reader to [5–9].

## HYBRID MODELS: PROMISES AND CHALLENGES

In general, the bottom-up approach provides a biologically plausible but functionally limited perspective, whereas the top-down approach offers functional performance at the potential expense of biological realism. Moreover, both approaches tend to develop models using a homogeneous implementation framework, usually combining all components of a brain model in a single codebase. This implies that translating the insights gleaned from the plethora of existing models of circumscribed brain structures or functions, which may have been implemented using vastly different tools and programming languages, requires their reimplementation within the specific framework of a particular integrative brain model. Hybrid models offer a potential solution to some of the above problems: they make it possible to blend biological plausibility and functional performance and support the direct integration of existing model implementations into a unified system. This is not only more efficient, but also provides a testing ground wherein models of circumscribed brain areas or cognitive functions can be evaluated within a holistic context. This provides a benchmark for such models as they need to demonstrate their ability to interact effectively with other components. A flexible, plug-and-play integration approach additionally supports continuous model refinement and comparative hypothesis testing. These considerations were a significant impetus behind the establishment of Work Package 3 (WP3) ‘Adaptive networks for cognitive architectures: from advanced learning to neurorobotics and neuromorphic applications’ of the Human Brain Project [10]. In addition to developing brain models using both bottom-up and top-down approaches, a key objective of WP3 was to specify a practical approach for the development of hybrid integrative brain models that can efficiently accomplish real-world tasks in embodied and situated settings, while at the same time maintaining a high degree of biological realism.

The Work Package has dedicated significant effort to identifying and surmounting the challenges that arise from this objective. The primary challenge lies in effectively connecting components (e.g. models of circumscribed brain areas or cognitive functions) to create a coherent system. This is complicated by the often-observed heterogeneity of the components. First, components may be implemented using different programming languages and frameworks, potentially leading to dependency issues. Second, components exhibit varying levels of abstraction from biological detail to high-level neurocomputational principles. One issue this raises is that of numerical stability: components may employ different numerical methods with varying stability conditions and accuracy characteristics. Varying levels of abstraction also raise issues surrounding communication and synchronization between heterogeneous components, which might operate at different timescales. Special care needs to be taken when integrating components into the overarching architecture. Inputs, outputs and internal states of each component need to be clearly specified to interface components correctly with each other and potentially with a body and the external environment. For example, one might need to specify how to convert signals between spiking and rate neurons. These issues are exacerbated in a plug-and-play setting that should allow replacement of any component by an alternative realization. Replacing components can easily disrupt interfaces to other components and hence the functionality of the system as a whole.

## MODULAR-INTEGRATIVE MODELING

To overcome these challenges, WP3 adopted a modular-integrative modeling approach. Inspired by modular simulation frameworks utilized in other scientific domains (e.g. [11]), this approach uses containerization technologies to encapsulate each component as a module in an isolated environment. Modularization helps to avoid dependency issues and allows each component to employ numerical methods that operate under their optimal conditions without interfering with other components. Modularization further supports subtask decomposition and hence initialization, pre-training and fine-tuning of each component independently. Importantly, modules allow the combination of differentiated learning paradigms, such as a basal ganglia model with reinforcement learning connected to a cerebellar model with supervised learning. Implementation-wise, as in modular deep learning, components can thus be optimized while retaining their overall context to ensure that solutions meet global task requirements as well as constraints imposed by other components [12]. This is highly efficient and generalizes better than end-to-end optimization of the full model [13]. Finally, modularization also promotes embodiment and situatedness by including modules dedicated to body and environment simulations. The resulting ability of the overarching system to generate overt behavior can be exploited for further fine-tuning of model components through learning. However, ensuring compatibility between modules can impose restrictions on their design and functionality. It is therefore crucial that each module is developed with its role within the larger system in mind and that a uniform data exchange format and precise interface specifications are in place.

Communication between components is achieved through a message broker using a topic-based publish-subscribe pattern. Any component may publish information such as neuron spike times or neural network layer activations on a dedicated topic. Other components that require this information subscribe to this topic. This communication method provides loose coupling between components as they do not need to know about the existence of a particular component realization, but only about the topics they are interested in. This ensures that individual components can be updated, modified or replaced without needing extensive changes to the broader system [11]. Additionally, a publish-subscribe pattern allows integration of information generated by a subset of components into a global signal that individual components may, in turn, subscribe to in order to modulate their internal state. This facilitates incorporation of global processes such as attention, potentially mediated via hub structures like the thalamus [14]. However, ensuring that messages are received and processed in the correct order can be challenging. Modules may operate at different speeds, and without proper synchronization; this can lead to race conditions or outdated information being acted upon. To address potential synchronization issues, the modular-integrative modeling approach incorporates a specialized simulation coordinator.

This coordinator guarantees a unified framework for time management, while allowing each component to independently publish and subscribe to topics. The simulation coordinator regulates simulation cycles, with each cycle representing a fixed global simulation time step. The simulation coordinator signals the start of cycles, ensuring synchronization between components. Importantly, while there is a fixed global simulation time step, each component can have its own internal time step based on its numerical method’s stability and accuracy requirements [11]. The simulation coordinator also coordinates the overall set-up of the simulation, including the specification of initial conditions and simulation duration as well as of the timing, duration and magnitude of external inputs.

**Figure 1.**
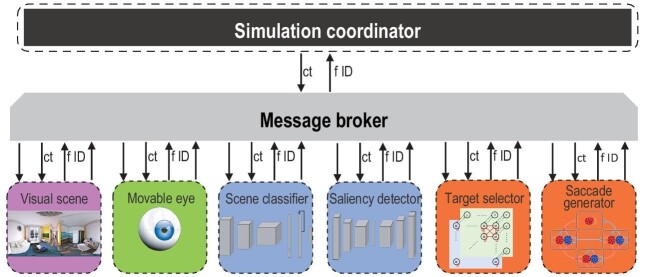
Example of a working implementation of the modular-integrative modeling approach devised within WP3 of the Human Brain Project. The example model performs saccades to facilitate scene understanding. The full system is divided into six modules: environment (visual scene), body (eye) and four components of an integrative brain model (scene classifier, saliency detector, target selector and saccade generator). All modules are containerized, as indicated by the dashed outlines. The colors of the modules indicate their types. Green and magenta indicate modules dedicated to body and environment simulations, respectively. Orange indicates modules associated with circumscribed brain components assumed to be implemented following a bottom-up approach. Blue indicates modules associated with circumscribed brain functions assumed to be implemented following a top-down approach. The simulation coordinator is indicated in dark gray, and the message broker in light gray. Note that the message broker is not containerized, but handles communication across containers. The simulation coordinator publishes the central time (to the ct topic) whenever it is incremented by a global time step. All modules subscribe to the ct topic, while also keeping track of their local time. Whenever the central time exceeds a module’s local time, the module performs a simulation for one global time step using a local time step appropriate for the particular module. When a module finishes its simulation for a specific global time step, it publishes its unique identifier (module ID) to the fID (finished ID) topic. The simulation coordinator subscribes to the fID topic to keep track of the modules that have finished their simulation for the current global time step. Once all modules have finished, the simulation coordinator updates the current time to initiate a new simulation cycle. This setup controls the simulation and is general to all hybrid models implemented using the modular-integrative approach. Additionally, any particular module may specify additional topics it either publishes or subscribes to. For example, the saliency detector publishes a saliency distribution over the visual scene and subscribes to snapshots from the scene that are published by the eye module. The target selection module subscribes to the saliency distribution and publishes the outcome of a spatial decision-making process as a desired eye position. The saccade generator, in turn, subscribes to the desired eye position and publishes the actual eye position, to which the eye subscribes. Finally, the scene classifier subscribes to snapshots from the scene and integrates information across snapshots to classify the overall scene.

The modular-integrative modeling approach lends itself to a multitude of use cases, including multisensory integration, multiscale dynamics and embodied cognition. One particular example of the latter is scene understanding, which operates under constraints imposed by the eye. The retina is characterized by a sharp decline of receptor density from the fovea to the periphery. This allows for high visual acuity, while maintaining a large field of view, but also necessitates the visual system to integrate high-resolution glimpses of a scene across various eye fixations. This process involves several perceptual and motor components, and the modular-integrative modeling approach is suitable for developing embodied models that efficiently incorporate and interface the necessary components. Figure 1 presents a possible implementation of a model that uses saccades to efficiently sample and classify a visual scene following the modular-integrative approach. For technical details of this example implementation, we refer the interested reader to https://github.com/ccnmaastricht/SSU.

## CONCLUSION

We argue that the modular-integrative modeling approach offers a promising avenue for constructing hybrid integrative brain models that enable the blending of biological plausibility and functional performance, hence providing the means to complement the bottom-up and top-down approaches. By incorporating global processes, specific structures and functions, this approach supports constructing models capturing the intricacies fundamental to diverse brain functions. It is crucial to emphasize that the modular aspect of the modular-integrative modeling approach is motivated by engineering considerations. It is distinct from (and does not necessarily require) the notion that the brain itself is neatly modular in its structure or function. We would further like to underscore the integrative nature of the approach, which is explicitly designed to counteract an overly compartmentalized perspective on the brain. It is interesting to consider that the brain itself must address the problem of integrating the contributions of different brain networks and functions, which show at least some degree of modular organization. While the proposed modular-integrative modeling approach was not primarily intended to model functional segregation and integration in the brain, we hope that it may catalyze a more comprehensive understanding of brain structure, dynamics and function, and of the interplay between the brain, the body and the environment.
